# Ferrocenylbutadiyne

**DOI:** 10.1107/S1600536809005522

**Published:** 2009-02-21

**Authors:** Victor N. Nemykin, Jason D. Dorweiler, Roman I. Subbotin

**Affiliations:** a Department of Chemistry & Biochemistry, University of Minnesota Duluth, 1039 University Drive, Duluth, MN 55812, USA

## Abstract

The title compound, [Fe(C_5_H_5_)(C_9_H_5_)], crystallizes in a form of a π–π-stacked assembly formed as a result of strong inter­molecular π–π inter­actions between (*a*) the triple bonds of two neighboring butadiyne substituents overlapping in a ‘head-to-tail’ fashion [characterized by C⋯C short contacts of 3.622 (5), 3.567 (6) and 3.556 (6) Å] and (*b*) the triple bonds of the butadiyne substituent and substituted cyclo­pendadiene ring of neighboring mol­ecules [C⋯C = 3.474 (5) and 3.492 (6) Å]. The linear butadiyne substituent has alternating C—C triple and single bonds, while the unsubstituted cyclo­penta­diene ring is slightly positionally disordered (although the structure reported here was solved as non-disordered) and retains a close to eclipsed conformation.

## Related literature

For the general synthesis and applications of substituted ferrocenes and related macrocycles, see: Fouda *et al.* (2007 ▶); Nemykin *et al.* (2001 ▶, 2007*a* ▶,*b* ▶, 2008 ▶); Stepnika (2008 ▶); Osakada *et al.* (2006 ▶). For the synthesis of the title compound, see: Yuan *et al.* (1993 ▶); Nemykin *et al.* (2007*c* ▶). For examples of the use of the title compound, see Bruce *et al.* (2004 ▶).
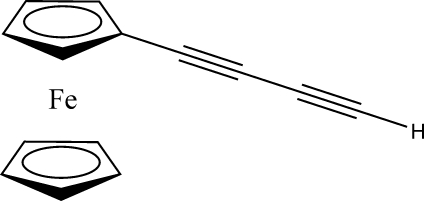


## Experimental

### 

#### Crystal data

[Fe(C_5_H_5_)(C_9_H_5_)]
*M* *_r_* = 234.08
Monoclinic, 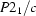

*a* = 7.9438 (16) Å
*b* = 10.332 (2) Å
*c* = 12.835 (3) Å
β = 97.01 (3)°
*V* = 1045.5 (4) Å^3^
*Z* = 4
Mo *K*α radiation
μ = 1.40 mm^−1^
*T* = 298 K
0.45 × 0.30 × 0.25 mm

#### Data collection

Rigaku AFC-7R diffractometer
Absorption correction: ψ scan (North *et al.*, 1968 ▶) *T*_min_ = 0.58, *T*_max_ = 0.70
2549 measured reflections
2411 independent reflections
2248 reflections with *I* > 2σ(*I*)
*R*_int_ = 0.052
3 standard reflections every 150 reflections intensity decay: none

#### Refinement

*R*[*F* ^2^ > 2σ(*F* ^2^)] = 0.048
*wR*(*F* ^2^) = 0.136
*S* = 1.08
2402 reflections
136 parameters
H-atom parameters constrained
Δρ_max_ = 0.52 e Å^−3^
Δρ_min_ = −0.47 e Å^−3^

### 

Data collection: *AFC-7R Diffractometer Control Software* (Rigaku/MSC, 1997 ▶); cell refinement: *WinAFC* (Rigaku/MSC, 2000 ▶); data reduction: *TEXSAN* (Rigaku/MSC, 2004 ▶); program(s) used to solve structure: *SIR92* (Altomare *et al.*, 1994 ▶); program(s) used to refine structure: *CRYSTALS* (Betteridge *et al.*, 2003 ▶); molecular graphics: *CAMERON* (Watkin *et al.*, 1996 ▶); software used to prepare material for publication: *CRYSTALS*.

## Supplementary Material

Crystal structure: contains datablocks global, I. DOI: 10.1107/S1600536809005522/hg2474sup1.cif

Structure factors: contains datablocks I. DOI: 10.1107/S1600536809005522/hg2474Isup2.hkl

Additional supplementary materials:  crystallographic information; 3D view; checkCIF report

## Figures and Tables

**Fig. 1.**
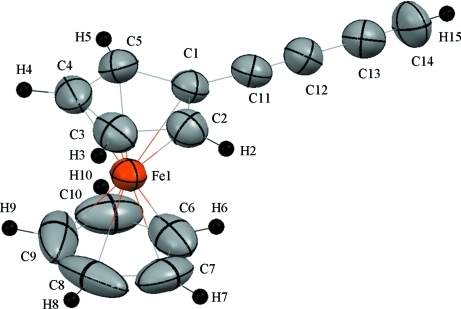
The title compound with displacement ellipsoids drawn at the 50% probability level. H atoms are shown as spheres of arbitary radius.

**Fig. 2.**
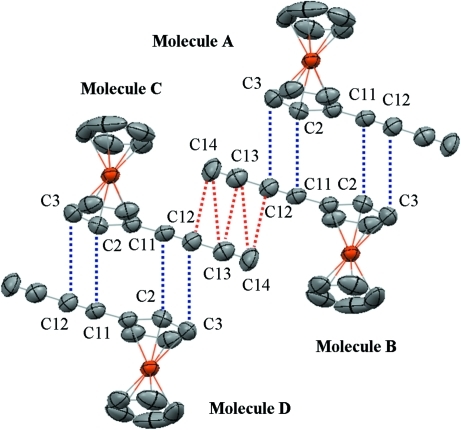
Intermolecular π-π interactions in the title compound. displacement ellipsoids drawn at the 50% probability level. Molecules located at: 1 - x, 3/2 + y, 3/2 - z (molecule A); 1 + x, 3/2 - y, 1/2 + z (molecule B); 1 - x, 1/2 + y, 3/2 - z (molecule C); and 1 + x, 1/2 - y, 1/2 + z (molecule D).

## supplementary crystallographic information

### Comment

Ferrocene derivatives have been useful as antitumor agents (Fouda *et al., *2007) and as electron transfer molecules. (Stepnika, 2008; Nemykin *et al.*, 2001, 2007*a*, 2007*b*, 2007*c*, 2008; Osakada *et al.*, 2006) The title compound represents a precursor for the preparation of butadiyne like dinuclear ferrocene molecules. (Bruce *et al.*, 2004, Yuan *et al.*, 1993).

There are a number of known structures of substituted ferrocenes (Stepnika, 2008, Nemykin *et al.*, 2007*a*, 2007*c*) but this is the first reported crystal structure of a butadiyne substituted ferrocene.

The molecule crystallizes as a π-π stacked assembly in the centrosymmetric monoclinic space group *P*2_1_/*c *. π-π stacked assembly formed as a result of strong intermolecular π-π interactions between (*a*) the triple bonds of two butadiyne substituents in molecules 'B' and 'C' (Figure 2) overlapping in 'head-to-tail' fashion and (*b*) the triple bonds of butadiyne substituents of a substituted cyclopendadiene ring along crystallographic *b* axis (Figure 2). Intermolecular π-π interactions between between the triple bonds of two butadiyne substituents (overlapping in 'head-to-tail' fashion) consists of three short contacts between C12 and C14 (3.622 (5) Å, *-x, 1 - y, 1 - z*), C13 and C14 (3.567 (6) Å, *-x, 1 - y, 1 - z*), and C13 and C13 (3.556 (6) Å, *-x, 1 - y, 1 - z*) carbon atoms of neighboring molecules. Intermolecular π-π interactions between between the triple bonds of butadiyne substituents and substituted cyclopentadiene ring of neighboring molecule can be characterized by two short contacts between C2 and C11 (3.474 (5) Å, *-x, -y, 1 - z*) and C3 and C12 (3.492 (6) Å, *-x, -y, 1 - z*) pairs of carbon atoms. The terminal H15 atom of the butadiyne substituent of one molecule is in close proximity to the H6 atom on the unsubstituted cyclopentadienyl ring of the other molecule. Although the unsubstituted cyclopentadiyne ring is, probably, disordered over two crystallographical positions (with disordered structure solution available from the authors on request), the unsubstituted cyclopentadiene ring retains close to eclipsed conformation of ferrocene subunit. In addition, the butadiyne substituent has alternating C—C triple and single bonds.

### Experimental

The title compound was obtained as by-product of the iodination reaction of ferrocenylacetylene (Nemykin *et al.*, 2007*c*). Melting point (81 *^o^*C, *dec.*). ^1^H NMR (CDCl_3_, tms, p.p.m.): 4.29, 5H, Cp; 4.63, 2H, α-Cp; 4.39, 2H, β-Cp; 2.39, 1H, butadiyne C—H. ^13^C (CDCl_3_, tms, p.p.m.): 71.0, Cp; 60.1, α-Cp; 70.6, β-Cp; 73.5, *i*-Cp; 66.9, ≡C—H; 70.4, C≡C—H; 71.2, Cp—C≡C-; 81.6, Cp-C≡C–). NMR spectra are similar to those reported earlier (Yuan *et al.*,1993)*.*

### Refinement

All cyclopentadienyl H atoms positioned geometrically, while the terminal butadiyne H atom was located on a Fourier map. The H atoms were initially refined with soft restraints on the bond lengths and angles to regularize their geometry (C(Ferrocene) - H 0.93; ≡C—H 0.82 Å) and *U*_iso_(H) (in the range 1.2–1.5 times *U*_eq_ of the parent atom) using default procedure available in Crystals for Windows software (Betteridge *et al.*, 2003). After this the positions were refined with riding constraints.

The difference between the number of independent reflections (2411) and those included in the refinement (2402) is originate from the filter used by Crystals for Windows software. The filter uses (sin theta/lambda)^2^ at least 0.0100 cutoff in order to eliminate reflections that may be poorly measured in the vicinity of the beam stop.

### Crystal data

**Table:**

[Fe(C_5_H_5_)(C_9_H_5_)]	*F*(000) = 480
*M**_r_* = 234.08	*D*_x_ = 1.487 Mg m^−^^3^
Monoclinic, *P*2_1_/*c*	Mo *K*α radiation, λ = 0.71073 Å
Hall symbol: -P 2ybc	Cell parameters from 25 reflections
*a* = 7.9438 (16) Å	θ = 15–18°
*b* = 10.332 (2) Å	µ = 1.40 mm^−^^1^
*c* = 12.835 (3) Å	*T* = 298 K
β = 97.01 (3)°	Block, brown
*V* = 1045.5 (4) Å^3^	0.45 × 0.30 × 0.25 mm
*Z* = 4	

### Data collection

**Table:**

Rigaku AFC-7R diffractometer	*R*_int_ = 0.052
graphite	θ_max_ = 27.5°, θ_min_ = 2.5°
ω/2θ scans	*h* = −10→10
Absorption correction: ψ scan (North *et al.*, 1968)	*k* = −13→0
*T*_min_ = 0.58, *T*_max_ = 0.70	*l* = 0→16
2549 measured reflections	3 standard reflections every 150 reflections
2411 independent reflections	intensity decay: 0.00%
2248 reflections with *I* > 2σ(*I*)	

### Refinement

**Table:**

Refinement on *F*^2^	Primary atom site location: structure-invariant direct methods
Least-squares matrix: full	Hydrogen site location: inferred from neighbouring sites
*R*[*F*^2^ > 2σ(*F*^2^)] = 0.048	H-atom parameters constrained
*wR*(*F*^2^) = 0.136	Method = Modified Sheldrick *w* = 1/[σ^2^(*F*^2^) + (0.07*P*)^2^ + 0.99*P*], where *P* = [max(*F*_o_^2^,0) + 2*F*_c_^2^]/3
*S* = 1.08	(Δ/σ)_max_ = 0.0003
2402 reflections	Δρ_max_ = 0.52 e Å^−^^3^
136 parameters	Δρ_min_ = −0.47 e Å^−^^3^
0 restraints	

### Fractional atomic coordinates and isotropic or equivalent isotropic displacement parameters (Å^2^)

**Table:**

	*x*	*y*	*z*	*U*_iso_*/*U*_eq_	
Fe1	0.29866 (5)	0.10003 (4)	0.31022 (3)	0.0488	
C1	0.1074 (4)	0.0794 (3)	0.4007 (3)	0.0518	
C2	0.2591 (4)	0.0175 (3)	0.4488 (3)	0.0575	
C3	0.3047 (5)	−0.0793 (3)	0.3799 (3)	0.0668	
C4	0.1850 (6)	−0.0782 (3)	0.2897 (3)	0.0710	
C5	0.0628 (4)	0.0191 (4)	0.3004 (3)	0.0622	
C6	0.3496 (7)	0.2904 (4)	0.2979 (5)	0.0866	
C7	0.4938 (7)	0.2257 (5)	0.3366 (4)	0.0928	
C8	0.5285 (7)	0.1368 (5)	0.2642 (8)	0.1210	
C9	0.4034 (14)	0.1459 (8)	0.1794 (5)	0.1336	
C10	0.2955 (7)	0.2426 (7)	0.2031 (5)	0.1036	
C11	0.0220 (4)	0.1842 (3)	0.4417 (3)	0.0543	
C12	−0.0531 (4)	0.2707 (4)	0.4767 (3)	0.0594	
C13	−0.1411 (5)	0.3705 (4)	0.5177 (3)	0.0665	
C14	−0.2128 (5)	0.4495 (4)	0.5504 (4)	0.0755	
H2	0.3169	0.0377	0.5142	0.0693*	
H3	0.3970	−0.1349	0.3926	0.0865*	
H4	0.1871	−0.1323	0.2320	0.0850*	
H5	−0.0298	0.0394	0.2515	0.0744*	
H6	0.2992	0.3561	0.3327	0.1061*	
H7	0.5582	0.2387	0.4013	0.1128*	
H8	0.6195	0.0796	0.2682	0.1788*	
H9	0.3901	0.0972	0.1180	0.1600*	
H10	0.2000	0.2722	0.1607	0.1248*	
H15	−0.2661	0.5084	0.5739	0.0916*	

### Atomic displacement parameters (Å^2^)

**Table:**

	*U*^11^	*U*^22^	*U*^33^	*U*^12^	*U*^13^	*U*^23^
Fe1	0.0479 (3)	0.0438 (3)	0.0572 (3)	−0.00690 (17)	0.01690 (19)	−0.00211 (17)
C1	0.0481 (15)	0.0538 (16)	0.0560 (16)	−0.0080 (13)	0.0158 (13)	−0.0011 (13)
C2	0.0606 (18)	0.0546 (17)	0.0591 (17)	−0.0015 (15)	0.0146 (14)	0.0087 (14)
C3	0.070 (2)	0.0454 (17)	0.090 (3)	0.0012 (15)	0.029 (2)	0.0067 (17)
C4	0.084 (3)	0.0535 (18)	0.083 (3)	−0.0227 (18)	0.038 (2)	−0.0177 (17)
C5	0.0541 (17)	0.069 (2)	0.0638 (18)	−0.0216 (16)	0.0107 (14)	−0.0091 (16)
C6	0.099 (3)	0.0441 (18)	0.128 (4)	−0.008 (2)	0.060 (3)	0.005 (2)
C7	0.082 (3)	0.099 (4)	0.093 (3)	−0.051 (3)	−0.003 (2)	0.017 (3)
C8	0.085 (4)	0.068 (3)	0.229 (8)	0.006 (3)	0.096 (5)	0.031 (4)
C9	0.206 (8)	0.120 (5)	0.093 (4)	−0.098 (5)	0.095 (5)	−0.043 (4)
C10	0.079 (3)	0.125 (4)	0.103 (4)	−0.035 (3)	−0.005 (3)	0.061 (4)
C11	0.0476 (16)	0.0597 (18)	0.0577 (17)	−0.0082 (14)	0.0149 (13)	0.0007 (14)
C12	0.0527 (17)	0.064 (2)	0.0631 (19)	−0.0020 (15)	0.0138 (14)	−0.0029 (15)
C13	0.058 (2)	0.073 (2)	0.070 (2)	−0.0079 (18)	0.0147 (17)	−0.0011 (18)
C14	0.073 (2)	0.066 (2)	0.091 (3)	0.0128 (19)	0.028 (2)	−0.018 (2)

### Geometric parameters (Å, °)

**Table:**

Fe1—C1	2.032 (3)	C4—C5	1.416 (6)
Fe1—C2	2.031 (3)	C4—H4	0.930
Fe1—C3	2.056 (3)	C5—H5	0.930
Fe1—C4	2.054 (3)	C6—C7	1.366 (7)
Fe1—C5	2.042 (3)	C6—C10	1.335 (8)
Fe1—C6	2.018 (4)	C6—H6	0.930
Fe1—C7	2.019 (4)	C7—C8	1.359 (8)
Fe1—C8	2.023 (4)	C7—H7	0.930
Fe1—C9	2.019 (4)	C8—C9	1.384 (10)
Fe1—C10	2.013 (4)	C8—H8	0.930
C1—C2	1.436 (5)	C9—C10	1.375 (10)
C1—C5	1.435 (5)	C9—H9	0.930
C1—C11	1.413 (5)	C10—H10	0.930
C2—C3	1.412 (5)	C11—C12	1.192 (5)
C2—H2	0.930	C12—C13	1.385 (5)
C3—C4	1.406 (6)	C13—C14	1.107 (5)
C3—H3	0.930	C14—H15	0.820
			
C1—Fe1—C2	41.37 (14)	Fe1—C2—H2	125.7
C1—Fe1—C3	68.68 (14)	C3—C2—H2	126.0
C2—Fe1—C3	40.40 (15)	C2—C3—Fe1	68.87 (19)
C1—Fe1—C4	68.39 (14)	C2—C3—C4	108.1 (3)
C2—Fe1—C4	67.87 (16)	Fe1—C3—C4	69.9 (2)
C3—Fe1—C4	40.00 (18)	C2—C3—H3	125.8
C1—Fe1—C5	41.25 (13)	Fe1—C3—H3	127.5
C2—Fe1—C5	69.11 (15)	C4—C3—H3	126.1
C3—Fe1—C5	68.30 (16)	C3—C4—Fe1	70.1 (2)
C4—Fe1—C5	40.45 (16)	C3—C4—C5	109.2 (3)
C1—Fe1—C6	108.53 (16)	Fe1—C4—C5	69.31 (19)
C2—Fe1—C6	122.10 (19)	C3—C4—H4	125.2
C3—Fe1—C6	156.7 (2)	Fe1—C4—H4	126.1
C4—Fe1—C6	162.4 (2)	C5—C4—H4	125.6
C5—Fe1—C6	125.9 (2)	C1—C5—C4	107.3 (3)
C1—Fe1—C7	125.7 (2)	C1—C5—Fe1	69.02 (17)
C2—Fe1—C7	108.70 (18)	C4—C5—Fe1	70.2 (2)
C3—Fe1—C7	122.0 (2)	C1—C5—H5	126.5
C4—Fe1—C7	156.2 (2)	C4—C5—H5	126.1
C5—Fe1—C7	162.5 (2)	Fe1—C5—H5	126.4
C1—Fe1—C8	162.0 (3)	Fe1—C6—C7	70.2 (2)
C2—Fe1—C8	125.2 (3)	Fe1—C6—C10	70.4 (3)
C3—Fe1—C8	108.8 (2)	C7—C6—C10	108.2 (5)
C4—Fe1—C8	122.0 (2)	Fe1—C6—H6	124.8
C5—Fe1—C8	156.0 (3)	C7—C6—H6	125.0
C1—Fe1—C9	155.8 (4)	C10—C6—H6	126.7
C2—Fe1—C9	161.8 (4)	C6—C7—Fe1	70.2 (2)
C3—Fe1—C9	125.7 (3)	C6—C7—C8	108.3 (5)
C4—Fe1—C9	108.88 (19)	Fe1—C7—C8	70.5 (3)
C5—Fe1—C9	120.9 (3)	C6—C7—H7	126.8
C1—Fe1—C10	121.2 (2)	Fe1—C7—H7	124.8
C2—Fe1—C10	156.2 (3)	C8—C7—H7	124.9
C3—Fe1—C10	162.7 (3)	Fe1—C8—C7	70.2 (3)
C4—Fe1—C10	126.9 (2)	Fe1—C8—C9	69.8 (3)
C5—Fe1—C10	108.81 (18)	C7—C8—C9	107.8 (5)
C6—Fe1—C7	39.6 (2)	Fe1—C8—H8	126.0
C6—Fe1—C8	66.3 (2)	C7—C8—H8	127.8
C7—Fe1—C8	39.3 (3)	C9—C8—H8	124.4
C6—Fe1—C9	66.4 (2)	C8—C9—Fe1	70.1 (3)
C7—Fe1—C9	66.6 (2)	C8—C9—C10	106.5 (5)
C8—Fe1—C9	40.0 (3)	Fe1—C9—C10	69.8 (3)
C6—Fe1—C10	38.7 (2)	C8—C9—H9	128.7
C7—Fe1—C10	65.8 (2)	Fe1—C9—H9	124.2
C8—Fe1—C10	66.4 (2)	C10—C9—H9	124.8
C9—Fe1—C10	39.9 (3)	C9—C10—Fe1	70.3 (3)
Fe1—C1—C2	69.28 (18)	C9—C10—C6	109.3 (5)
Fe1—C1—C5	69.73 (18)	Fe1—C10—C6	70.9 (3)
C2—C1—C5	107.2 (3)	C9—C10—H10	126.6
Fe1—C1—C11	124.0 (2)	Fe1—C10—H10	125.6
C2—C1—C11	126.6 (3)	C6—C10—H10	124.1
C5—C1—C11	126.1 (3)	C1—C11—C12	178.5 (3)
C1—C2—Fe1	69.34 (18)	C11—C12—C13	179.5 (4)
C1—C2—C3	108.2 (3)	C12—C13—C14	179.3 (5)
Fe1—C2—C3	70.7 (2)	C13—C14—H15	179.3
C1—C2—H2	125.8		
